# Endotoxemia related to cardiopulmonary bypass is associated with increased risk of infection after cardiac surgery

**DOI:** 10.1186/cc9427

**Published:** 2011-03-11

**Authors:** DJ Klein, F Briet, R Nisenbaum, A Romaschin, C Mazer

**Affiliations:** 1 St Michael's Hospital, Toronto, Canada

## Introduction

The purpose of this study was to examine the prevalence of endotoxemia-supported aortocoronary bypass grafting surgery (ACB), using the endotoxin activity assay (EAA), and to explore the association between endotoxemia and postoperative infection.

## Methods

The study was a single-center prospective observational study measuring EAA during the perioperative period for elective ACB. Blood samples were drawn at induction of anesthesia (T1), immediately prior to release of the aortic cross-clamp (T2), and on the first postoperative morning (T3). The primary outcome was the prevalence of endotoxemia. The secondary outcome was rate of postoperative infection. An EAA of <0.40 was interpreted as low, 0.41 to 0.59 as intermediate, and >0.60 as high.

## Results

**Figure 1:**
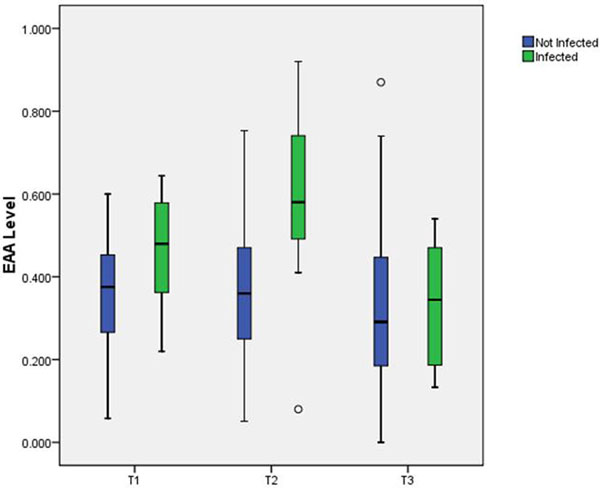
**Endotoxin levels in subjects with and without postoperative infections**.

Fifty-seven patients were enrolled and 54 patients were analyzable. The mean EAA at T1 was 0.38 ± 0.14, at T2 0.39 ± 0.18, and at T3 0.33 ± 0.18. At T2 only 13.5% of patients had an EAA in the high range. There was a positive correlation between EAA and the duration of cross-clamp (*P *= 0.02). Eight patients developed postoperative infections (14.6%). EAA at T2 was strongly correlated with the risk of postoperative infection (*P *= 0.02) as was the maximum EAA over the first 24 hours (*P *= 0.02). See Figure 1.

## Conclusions

High levels of endotoxin occurred less frequently during ACB than previously documented. However, endotoxemia is associated with a significantly increased risk of the development of postoperative infection - a complication associated with an over doubling of risk of death. Measuring endotoxin levels may provide a mechanism to identify and target a high-risk population.

